# Optimizing microbiome reference databases with PacBio full-length 16S rRNA sequencing for enhanced taxonomic classification and biomarker discovery

**DOI:** 10.3389/fmicb.2024.1485073

**Published:** 2024-11-25

**Authors:** Hyejung Han, Yoon Hee Choi, Si Yeong Kim, Jung Hwa Park, Jin Chung, Hee Sam Na

**Affiliations:** ^1^Department of Oral Microbiology, School of Dentistry, Pusan National University, Yangsan, Republic of Korea; ^2^Department of Internal Medicine, Dongnam Institute of Radiological and Medical Sciences, Busan, Republic of Korea

**Keywords:** oral microbiome, gut microbiome, PacBio, Illumina, next generation sequencing, reference database

## Abstract

**Background:**

The study of the human microbiome is crucial for understanding disease mechanisms, identifying biomarkers, and guiding preventive measures. Advances in sequencing platforms, particularly 16S rRNA sequencing, have revolutionized microbiome research. Despite the benefits, large microbiome reference databases (DBs) pose challenges, including computational demands and potential inaccuracies. This study aimed to determine if full-length 16S rRNA sequencing data produced by PacBio could be used to optimize reference DBs and be applied to Illumina V3-V4 targeted sequencing data for microbial study.

**Methods:**

Oral and gut microbiome data (PRJNA1049979) were retrieved from NCBI. DADA2 was applied to full-length 16S rRNA PacBio data to obtain amplicon sequencing variants (ASVs). The RDP reference DB was used to assign the ASVs, which were then used as a reference DB to train the classifier. QIIME2 was used for V3-V4 targeted Illumina data analysis. BLAST was used to analyze alignment statistics. Linear discriminant analysis Effect Size (LEfSe) was employed for discriminant analysis.

**Results:**

ASVs produced by PacBio showed coverage of the oral microbiome similar to the Human Oral Microbiome Database. A phylogenetic tree was trimmed at various thresholds to obtain an optimized reference DB. This established method was then applied to gut microbiome data, and the optimized gut microbiome reference DB provided improved taxa classification and biomarker discovery efficiency.

**Conclusion:**

Full-length 16S rRNA sequencing data produced by PacBio can be used to construct a microbiome reference DB. Utilizing an optimized reference DB can increase the accuracy of microbiome classification and enhance biomarker discovery.

## Introduction

The study of the human microbiome serves several important purposes, encompassing a wide range of medical objectives. It can help identify the imbalances associated with various diseases, such as inflammatory bowel disease (IBD), diabetes, obesity, and cardiovascular diseases (Li et al., 2016; Jie et al., 2017; Haneishi et al., 2023). Studying the human microbiome enables the identification of microbial biomarkers for early diagnosis, prognosis, and disease monitoring (Boppana et al., 2024). It can also detect pathogenic microorganisms that may contribute to infections or chronic diseases (Dong et al., 2024). Additionally, microbiome profiles can be used to predict the risk of developing various diseases (He et al., 2024).

The development of sequencing platforms has revolutionized the study of microbial communities. The gold standard for studying the taxonomic composition of a bacterial community is the sequencing of the 16S rRNA gene (Woese and Fox, 1977). 16S rRNA gene is around 1,500 bp long and has 9 variable regions that collect the main evolutionary changes among microbial taxa (Stackebrandt and Goebel, 1994). Compared to whole genome sequencing (WGS), 16S rRNA sequencing is more cost-effective, making it accessible for large-scale studies and routine analysis. Also, the methodologies for 16S rRNA gene amplification, sequencing, and analysis are well-established, providing a robust framework for researchers (Bolyen et al., 2019).

There are extensive public databases (DBs) (e.g., SILVA, Greengenes, RDP) for reference, facilitating accurate taxonomic assignment (Wang et al., 2007; Quast et al., 2013; DeSantis et al., 2006; Cole et al., 2014). While large microbiome reference DBs offer numerous advantages, such as improved resolution and comprehensive taxonomic coverage, they also come with certain disadvantages. Large DBs require significant computational power and memory for searching and aligning sequences. The sheer volume of data in large reference DBs can lead to longer processing times for sequence alignment and classification (Baker, 2010). Large DBs often contain redundant sequences or highly similar entries, which can complicate classification and lead to ambiguities in taxonomic assignments. The likelihood of incorporating erroneous or misannotated sequences increases, which can reduce the accuracy of taxonomic classifications and potentially lead to false conclusions (Sczyrba et al., 2017). Thus, selecting an optimal reference DB is crucial for microbiome studies. An optimal reference DB ensures accurate identification and classification of microbial taxa, reducing the chances of misidentification or ambiguous results, which is essential for understanding the true composition of the microbiome (Monika Balvočiūtė et al., 2017).

The oral and gut microbiome are the two most commonly studied human microbiome. Studying the oral microbiome has several advantages over the gut microbiome. The oral microbiome typically has a lower microbial diversity compared to the gut microbiome (Human Microbiome Project C, 2012). Also, the oral microbiome has been extensively studied, resulting in well-characterized reference DBs such as Human Oral Microbiome Database (HOMD) specifically tailored for oral bacteria, which facilitates more accurate taxonomic assignment (Dewhirst et al., 2010).

For microbiome study, Illumina platform has been widely used. Illumina platforms can sequence millions of reads per run, making it suitable for large-scale studies. The cost of sequencing per base is relatively low, and it provides high accuracy with low error rates. However, typical sequencing read length is rather short (2 × 300 bps), which cannot cover the full-length of the 16S rRNA gene, which could lead to potential misclassification or ambiguous taxonomic assignment (Satam et al., 2023). Pacbio and Nanopore can provide long read sequences to overcome this limitation. Especially, Pacbio system can provide improved sequencing quality with the development of circular consensus sequencing (CCS) protocols which generates highly accurate long high-fidelity reads, also known as HiFi reads (Wenger et al., 2019). Callahan et al. demonstrated that Pacbio HiFi could offer a single-nucleotide resolution by DADA2 approach based on Amplicon Sequence Variant (ASV) classification (Callahan et al., 2019). Thus, we hypothesized that full-length 16S rRNA sequencing data produced by PacBio could be used to optimize reference database in human microbiome studies.

Recently, there have been several studies that simultaneously utilized PacBio and Illumina platform for microbiome study and compared their performance (Buetas et al., 2024; Souza et al., 2023; Katiraei et al., 2022). Especially, She et al. have performed microbiome analysis on 53 sites of 7 surface human organs using both Illumina V3-V4 short read sequencing and Pacbio 16S rRNA full-length sequencing (She et al., 2024). In this study, we tested if full-length 16S rRNA sequencing data produced by Pacbio could be used to serve as a reference DB and compared it with commonly used reference DB (e.g., HOMD) for coverage and classification performance against V3-V4 short read sequencing data. To validate the method, we applied the optimization method to gut microbiome data. Optimized reference DB was constructed with ASVs, and it was compared to SILVA and Greengene reference DB in taxonomy assignment and biomarker discovery against Illumina V3-V4 short read sequencing data.

## Materials and methods

### Data

The raw sequencing data have been retrieved from NCBI GenBank BioProject ID PRJNA1049979. For oral microbiome study, 32 samples were sequenced by Pacbio and 198 samples were sequenced by Illumina platform. For gut microbiome study, 45 samples were sequenced by Pacbio and 128 samples were sequenced by Illumina. Summary of sampling site and sample number is shown in Tables 1, 2.

**Table 1:** Summary of sampling site, sample number and read counts during PacBio data preprocessing.

Platform	Organ	Site	Sample (*n*)	Input	Primers	Filtered	Denoised	Non-chimera
PacBio	Oral	Oral (pooled)	32	12,951 ± 910	9,945 ± 1,080	8,684 ± 1,781	8,098 ± 1,910	7,954 ± 1,895
	Large Intestine	ANAL	14	12,911 ± 1,029	10,596 ± 977	10,167 ± 1,412	9,789 ± 1,331	9,644 ± 1,301
	Small Intestine	IIC	10	13,475 ± 1,048	9,548 ± 1,102	8,805 ± 1,439	8,261 ± 1,550	7,892 ± 1,366
		IICP	7	12,753 ± 751	9,641 ± 1,216	8,361 ± 1,253	7,542 ± 1,596	6,959 ± 1,476
		JEJ100	14	12,734 ± 616	9,701 ± 836	7,316 ± 1,380	6,555 ± 1,328	6,380 ± 1,321

**Table 2:** Summary of sampling site, sample number and read counts during Illumina data preprocessing.

Platform	Organ	Site	Sample (*n*)	Input	Primers	Filtered	Denoised	Non-chimera
Illumina	Oral	LC	33	89,809 ± 10,028	68,550 ± 9,666	66,691 ± 9,475	22,814 ± 10,563	5,418 ± 1,953
		LL	33	96,880 ± 12,073	65,875 ± 9,331	58,444 ± 9,685	17,969 ± 10,157	6,117 ± 2,832
		LM	33	89,682 ± 7,948	70,752 ± 8,397	66,226 ± 9,156	23,383 ± 11,379	6,803 ± 2,598
		RC	33	88,192 ± 9,343	65,483 ± 6,869	63,748 ± 6,766	18,269 ± 9,126	3,552 ± 1,626
		UL	33	92,227 ± 11,036	66,759 ± 8,188	61,644 ± 8,331	17,002 ± 8,369	4,131 ± 1,818
		UM	33	100,157 ± 13,446	67,975 ± 10,536	63,106 ± 9,812	20,821 ± 11,657	6,522 ± 3,475
	Large intestine	ANAL	33	88,919 ± 6,446	77,305 ± 6,658	74,090 ± 7,276	43,646 ± 12,858	8,647 ± 2,256
	Small intestin	IIC	31	97,218 ± 11,533	67,811 ± 9,166	65,068 ± 9,595	38,822 ± 13,853	7,271 ± 1,772
		IICP	33	90,417 ± 12,012	63,174 ± 11,567	60,082 ± 10,734	16,895 ± 13,204	4,465 ± 2,614
		JEJ100	31	96,860 ± 24,706	74,051 ± 14,430	62,612 ± 12,818	9,310 ± 8,573	3,166 ± 1,791

### Bioinformatic analysis, statistical analysis, and visualization

For PacBio 16S full-length sequencing data, DADA2 algorithm was applied to dereplicate the reads and filter chimeric sequences. The ASVs were taxonomically assigned using RDP DB. Rarefaction analyses were conducted by vagan package.

To run stand-alone Basic Local Alignment Search Tool (BLAST) tool kits for alignment statistics, blast reference DB was constructed with PacBio ASVs and eHOMD, respectively. BLAST was performed against Illumina V3-V4 short read sequencing data to determine the alignment score, length of nucleotide identity and percentage of identity.

Phylogenetic tree construction by using *align-to-tree-mafft-fasttree* implemented in QIIME2 and visualized using iTOL (Ivica Letunic et al., 2021). Trimming phylogenetic was performed using *drop.tip* in ape package.

For gut microbiome, ASVs from PacBio sequencing data was trimmed and was used to construct reference DB. For Illumina 16S V3-V4 sequencing data, raw paired-end reads of 16S rRNA gene sequence were quality-filtered and analyzed using QIIME2 software with default parameters (version 2023.9.0) (Hall and Beiko, 1849) and associated plugins. Microbial community analysis, including *α*-diversity and *β*-diversity, were calculated using phyloseq R package. α-Diversity was evaluated by Choa1 index and Shannon’s index. β-diversity was measured by Bray-Curtis distance, and principal coordinates analysis (PCoA) was used for ordination analysis. Bacterial taxonomy was determined by pre-trained Naive Bayes classifier using either Greengene DB, SILVA DB or optimized PacBio reference DB.

Differentially enriched microbes were analyzed using Linear discriminant analysis (LDA) Effect Size (LefSe) (Segata et al., 2011), a methodology for performing differential abundance analysis of microbiome data. LDA score over 3 were considered significant. The codes are available at http://doi.org/10.5281/zenodo.13937633.

## Results

### Analysis of oral microbiome data

A total of 569,845 reads from the 32 oral samples were generated by PacBio long read sequencing. The mean number of sequences per sample was 12,951 ± 910, and the average read length was 1,457.7 ± 18.2 (1,392 – 1,595). After removing sequencing errors and chimera, a total of 349,997 reads remained, for an average of 7,954 ± 1,894 reads per sample (Table 1).

**Figure 1:**
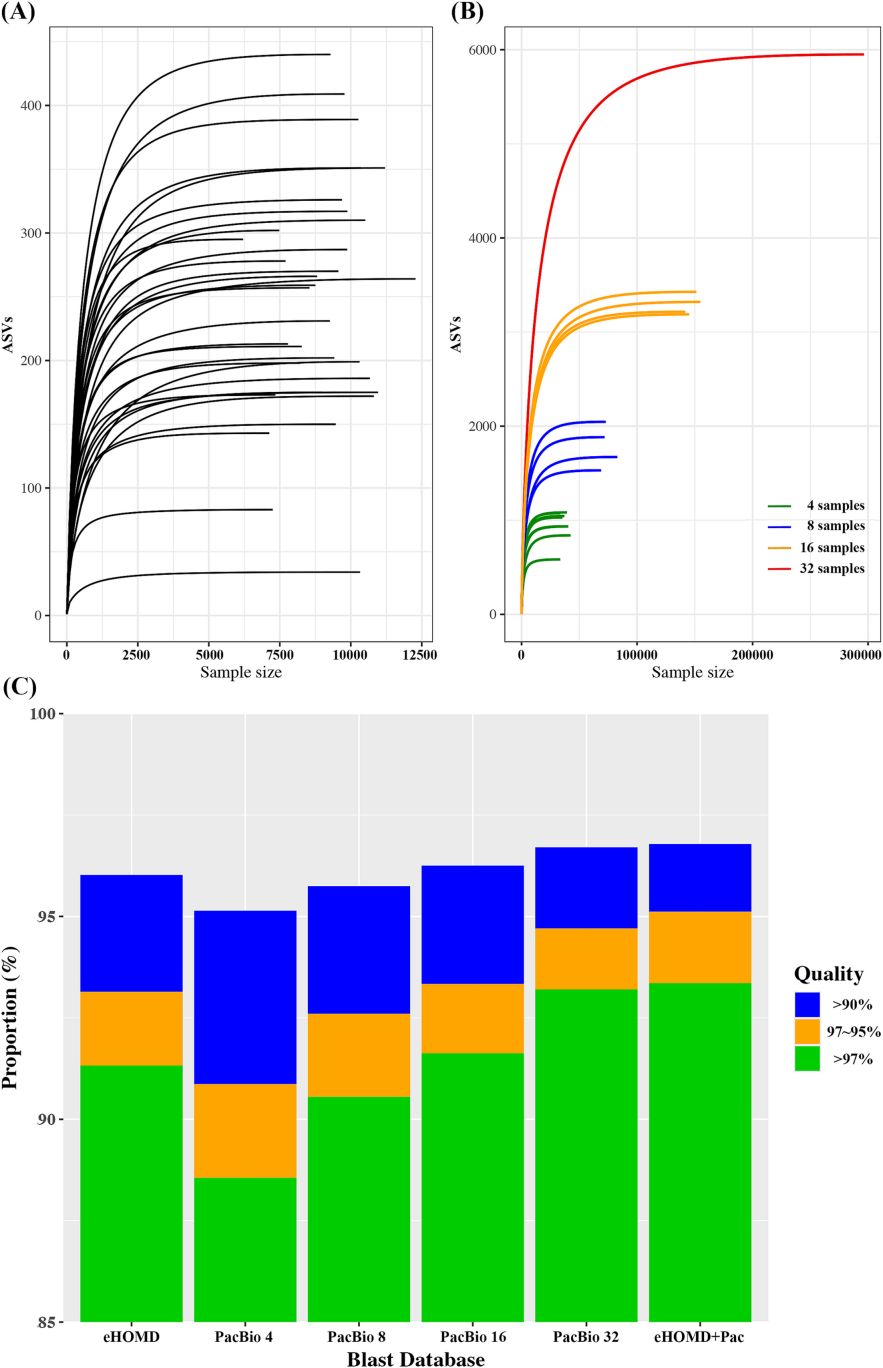
**(A)** Rarefaction curve for each oral sample. **(B)** Rarefaction curve for randomly combined oral samples. **(C)** Blast search result on Illunina V3-V4 oral microbiome data using various reference databases.

The average ASVs detected in each sample was 247.4 ± 91.0 (34–440). To assess the diversity and adequacy of sequencing depth, rarefaction curve was plotted for each sample. The rarefaction curve demonstrated good depth of coverage, leveling off at approximately 5,000 reads (Figure 1A). Since human microbiome is highly diverse and variable among individuals, we randomly combined oral samples to test if combining samples could increase the coverage. When 4, 8,16, and 32 samples were randomly combined, the average ASVs found in each combination was 940 ± 167, 1783 ± 228, 3267.5 ± 74, and 5,950, respectively. Thus, the number of ASVs detected was increased as the number of samples combined was increased (Figure 1B).

Although combining more samples produces a greater number of ASVs, it also increases the effort and budget required for the analysis. Therefore, determining an optimal number of samples should be essential. We constructed a BLAST reference DB with various combination of samples and compared the results against the eHOMD, a reference commonly used for oral microbiome analysis. For PacBio data, the proportion of successful BLAST searches increased with the number of ASVs in the DB. Comparing eHOMD and PacBio_4, which had a similar number of ASVs, the proportion of read counts with high identity (>97%) was significantly higher in eHOMD. The PacBio sample combination that showed comparable BLAST search performance to eHOMD was PacBio_16. Furthermore, PacBio_32, which had six times more ASVs than eHOMD, showed only a slight improvement (Figure 1C). Thus, using a DB with more ASVs did not necessarily result in the detection of higher identity.

To improve blast efficiency, we selected ASVs (+Pac) that showed high identity (>97%) by PacBio_32 while eHOMD showed less than 97% identity. Generally, species are clustered by sequence homology above 97% (Yarza et al., 2014). Since biomarkers are typically identified at the species level, we selected 97% as the specificity threshold. If the BLAST search results show high identity in both eHOMD and PacBio_32, this indicates a good match, regardless of the reference DB. However, if the BLAST search results show high identity in PacBio_32 but low identity (below 97%) in eHOMD, it suggests that the ASV in the PacBio_32 may serve as a better reference, which is not found in eHOMD. When eHOMD was combined with 130 ASVs (eHOMD+Pac), highest taxonomic assignment efficiency in BLAST searches was achieved compared to other DBs (Figure 1C).

### Phylogenetic tree-based optimization of PacBio ASVs

**Figure 2:**
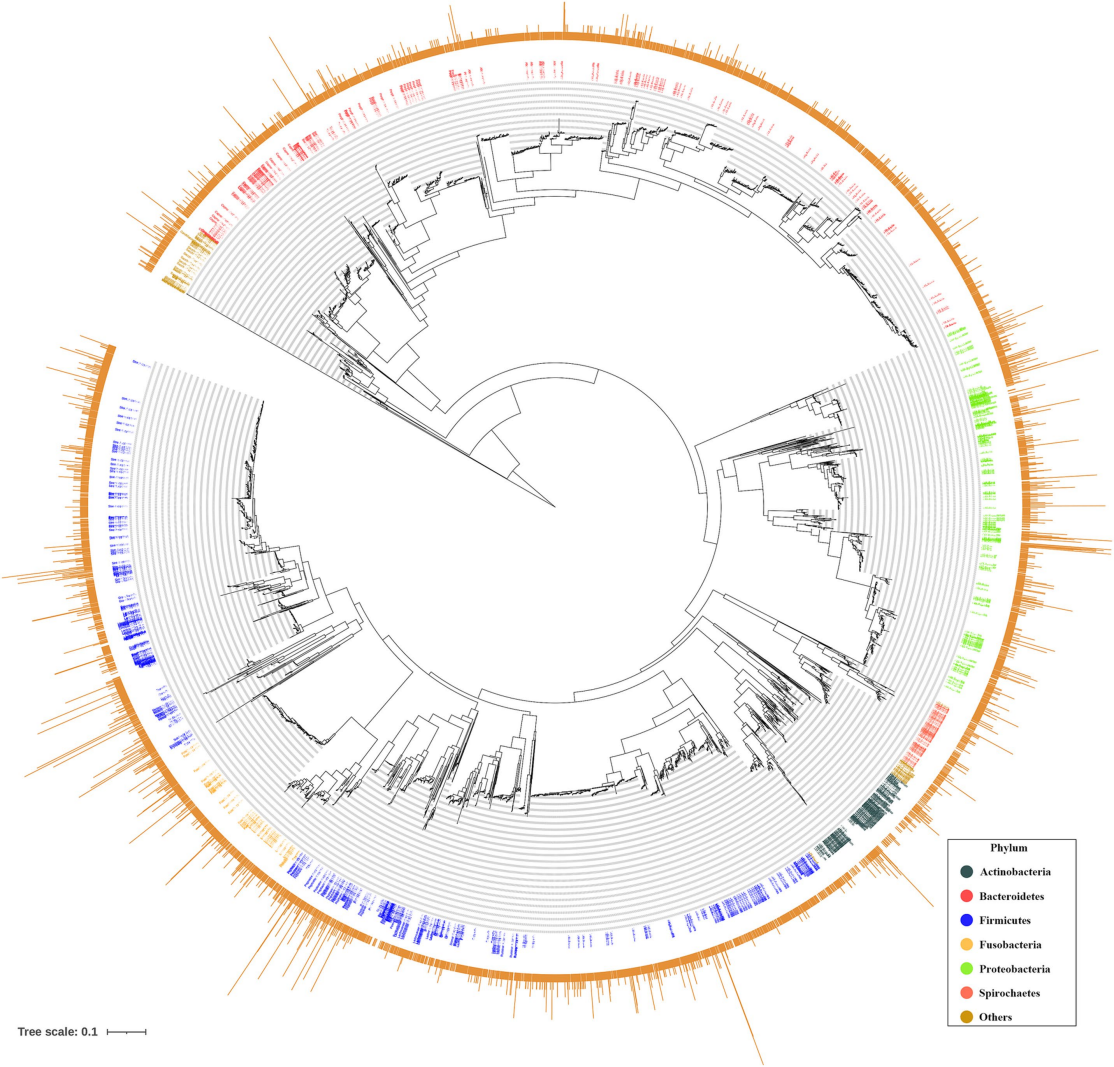
Phylogenetic trees of combined with eHOMD reference sequences and ASVs obtained from PacBio. In inner ring, colors represent phyla assigned by eHOMD. In outer ring, bar height represents number of oral samples present with the corresponding ASVs.

When a phylogenetic tree was constructed using PacBio_32 ASVs combined with eHOMD sequences, most of the trees included sequences from both DBs, suggesting that both DBs covered similar taxa (Figure 2).

**Figure 3:**
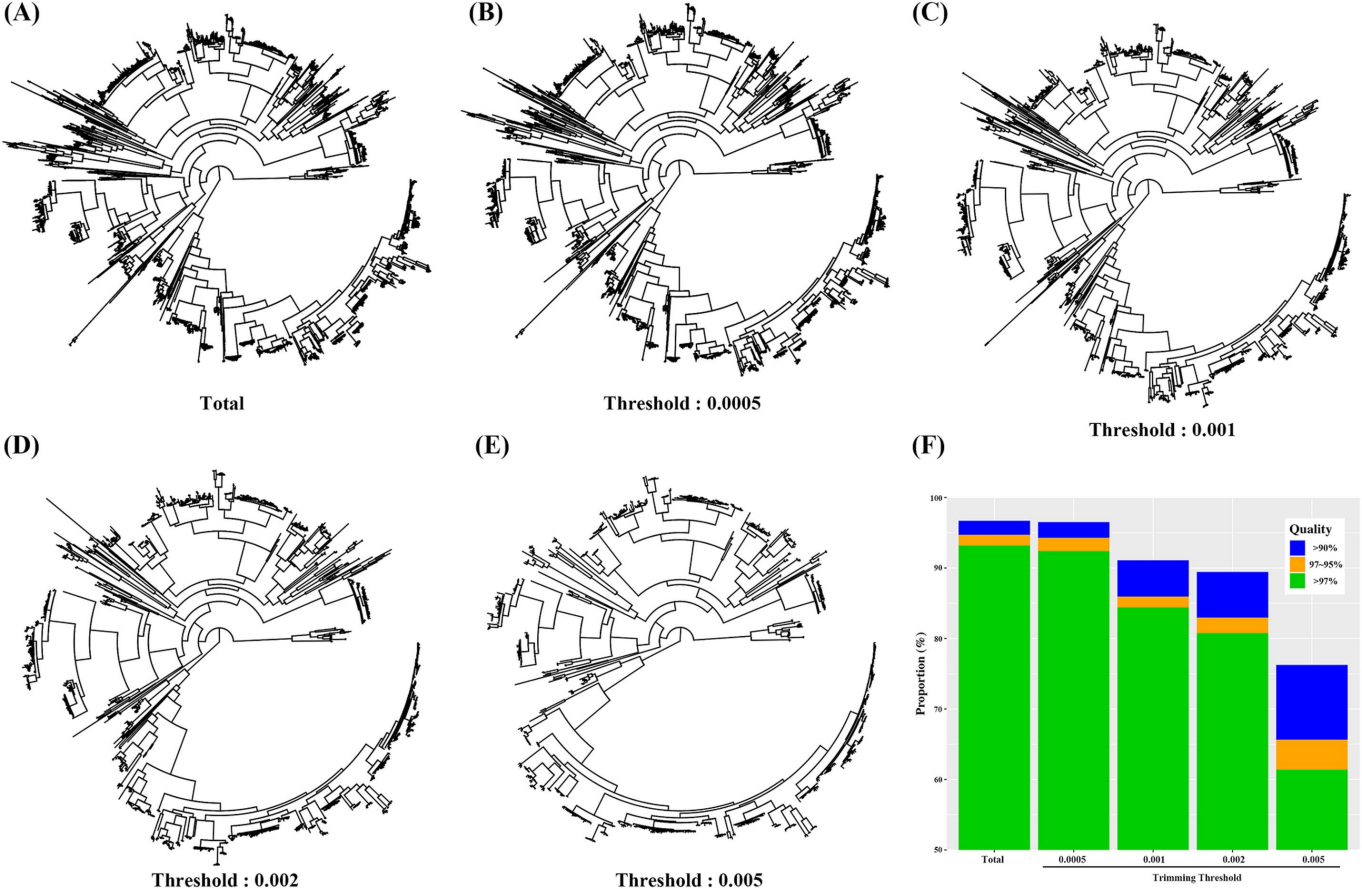
Phylogenetic trees of gut ASVs obtained from PacBio reads were trimmed with various thresholds. **(A)** Total ASVs, **(B)** threshold 0.0005, **(C)** threshold 0.001, **(D)** threshold 0.002, **(E)** threshold 0.001, **(F)** Blast search result on Illunina V3-V4 gut microbiome data using ASVs trimmed at various threshold as reference DB.

Given the substantial size difference between the PacBio ASVs and eHOMD, we sought to optimize the PacBio ASVs. To select representative sequences among similar ASVs, we employed the *drop_tip* function from the vegan package with various threshold to remove terminal branches (Figures 3A–E). After constructing the BLAST DB with ASVs trimmed with various thresholds, we performed BLAST searches against Illumina V3-V4 oral microbiome data. As the threshold value increased, the number of ASVs included in the BLAST DB decreased, and the proportion of read counts with high identity (>97%) also decreased. With a trimming threshold of 0.0005, the number of ASVs in the BLAST reference DB was reduced by approximately 50%, yet the BLAST search performance remained similar to the original ASVs (Figure 3F). This approach allows for the efficient optimization of DB size while maintaining taxonomic assignment accuracy.

### Analysis of gut microbiome data

Among various sampling sites in the gut, samples from small intestine (IIC, IICP, and JEJ100) and large intestine (ANAL) were selected for the analysis in this study. A total of 583,036 reads from the 45 gut samples were generated by PacBio long read sequencing. The mean number of sequences per sample was 12,956 ± 900. After removing sequencing errors and chimera, a total of 351,966 reads remained, for an average of 7,821 ± 1,879 reads per sample (Table 2). The PacBio reference DB was constructed by optimizing the ASVs based on oral microbiome results. After constructing the phylogenetic tree, tree tips were trimmed using a threshold of 0.0005. A total of 126 samples were tested from Illumina V3-V4 sequencing data.

**Figure 4:**
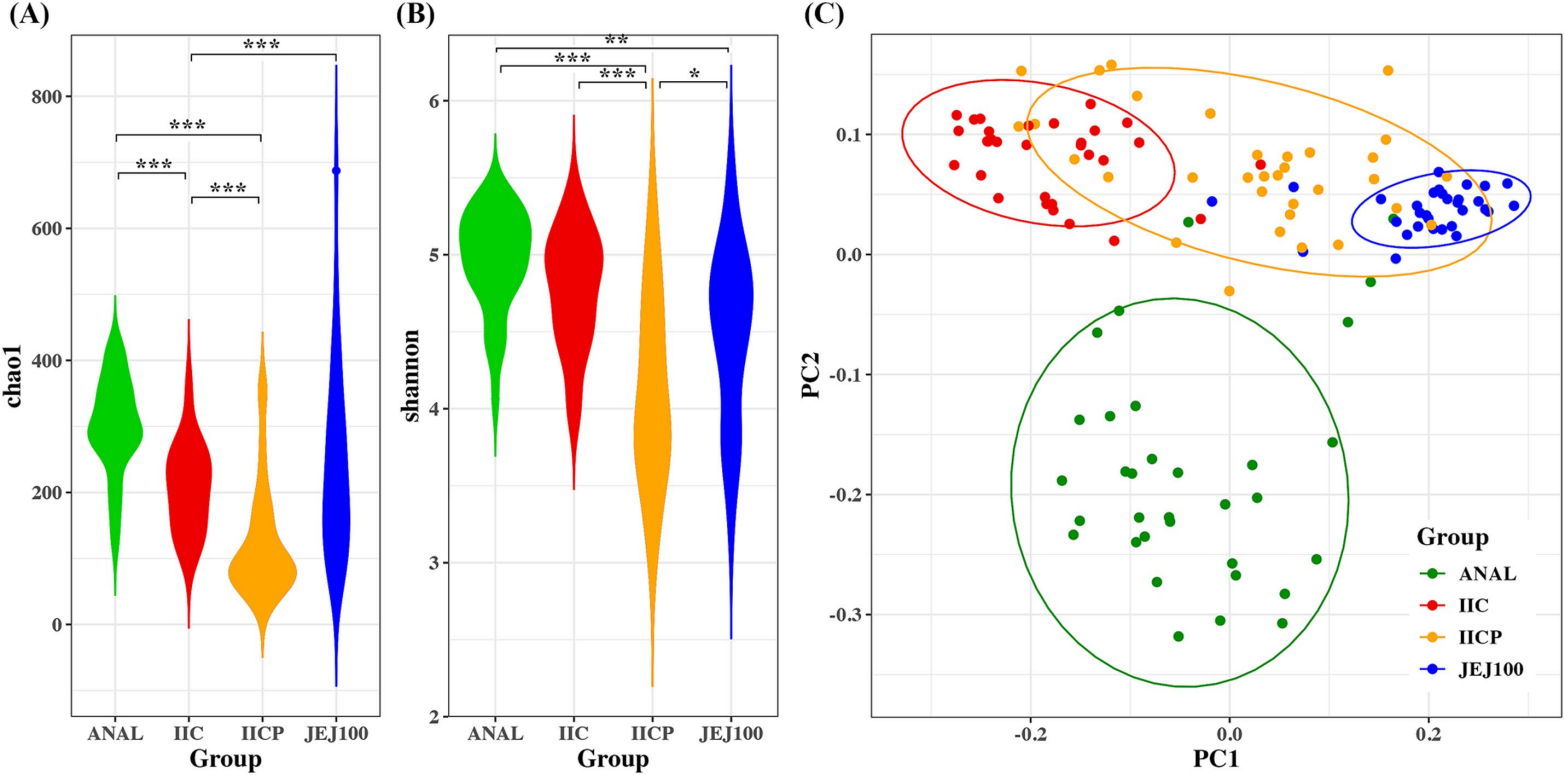
Bacterial community comparisons among gut sampling sites. Alpha diversity was used to describe the microbial richness and evenness within samples using the **(A)** Chao1 and **(B)** Shannon index. **(C)** Beta diversity of gut microbiome depending on sampling sites. Principal coordinate analysis (PCoA) of the Bray-Curtis distance was performed to determine the microbial community structure. *p < 0.05, **p < 0.01, ***p < 0.001.

Alpha diversity was measured to determine within microbiome diversity. The Chao1 index, reflecting richness, and Shannon index, reflecting evenness, were significantly different among gut sampling sites (Figures 4A,B). To compare bacterial community structure, beta-diversity analyses were performed on the corresponding samples. In the Bray Curtis-based principal coordinates analysis (PCoA), gut microbial community structure showed significant difference depending on the sampling sites (Figure 4C).

**Figure 5:**
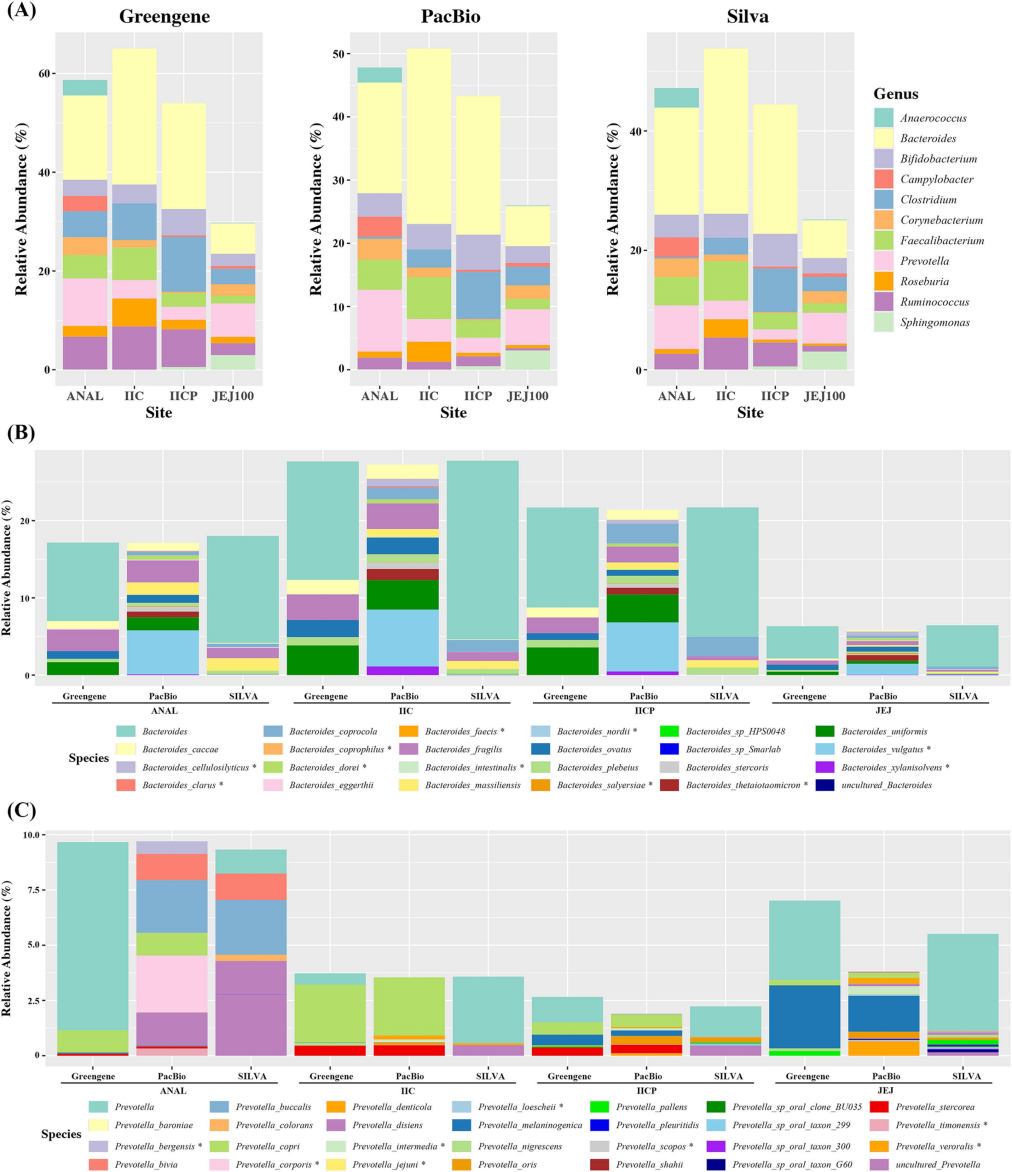
Average relative abundance of microbiome depending on various reference database. **(A)** Genus level, **(B)** *Bacteroides* at species level. **(C)** *Prevotella* at species level.

Each V3-V4 paired-reads were taxonomically assigned by pre-trained Naive Bayes classifier using either Greengene DB, SILVA DB or DB constructed by gut PacBio ASVs. At genus level, the overall relative abundance showed similar proportion regardless of the DB. However, there were some differences depending on the reference DB. The abundance of *Ruminococcus* was much higher in ANAL, IIC, and IICP using Greengene DB and while it showed low proportion using Pacbio DB. The abundance of *Clostridium* was much higher in ANAL samples using Greengene DB compared to other references (Figure 5A). In addition, when alpha diversity was measured at genus level, SILVA showed significantly higher indexes compared to PacBio and Greengene (Supplementary Figures S1A,C).

At the species level, we compared the abundance of *Bacteroides* and *Prevotella*. For *Bacteriodes*, Greengene and SILVA could not classify more than 50% to the species level and named them as *Bacteriodes*, while Pacbio distinguished most of the *Bacteroides* to the specific species. Moreover, some species were only found in PacBio. For example, *B. cellulosilyticsu, B. dorei, B. thetaiotaomicron* and *B. xylanisolvens* were assigned using Pacbio DB in all gut sampling sites, whereas they were not found in the other two DBs. Similarly, *B. clarus* was only found in Pacbio in IIC (Figure 5B). In *Prevotella*, there was some discrepancy in the proportion of the bacteria depending on the sampling site. The abundance of *Prevotella* was lower in IICP and JEJ compared to other reference DBs. However, Pacbio DB distinguished most of the *Prevotella* to the specific species, while Greengene and SILVA failed to assign to the specific species. Also, Pacbio was able to assign eight more *Prevotella* species. *P. bergensis, P. corporis* and *P. timinensis* were only found in ANAL. *P. intermedia* and *P. loescheii* were found in various small intestines (Figure 5C). In addition, when alpha diversity was measured at species level, PacBio showed significantly higher indexes compared to SILVA and Greengene (Supplementary Figures S1B,D). Taken together, an improvement in species assignment was observed when the PacBio DB was used across all four gut microbiome samples compared to the other two DBs.

### Species taxa comparison in depending on reference DB

**Figure 6:**
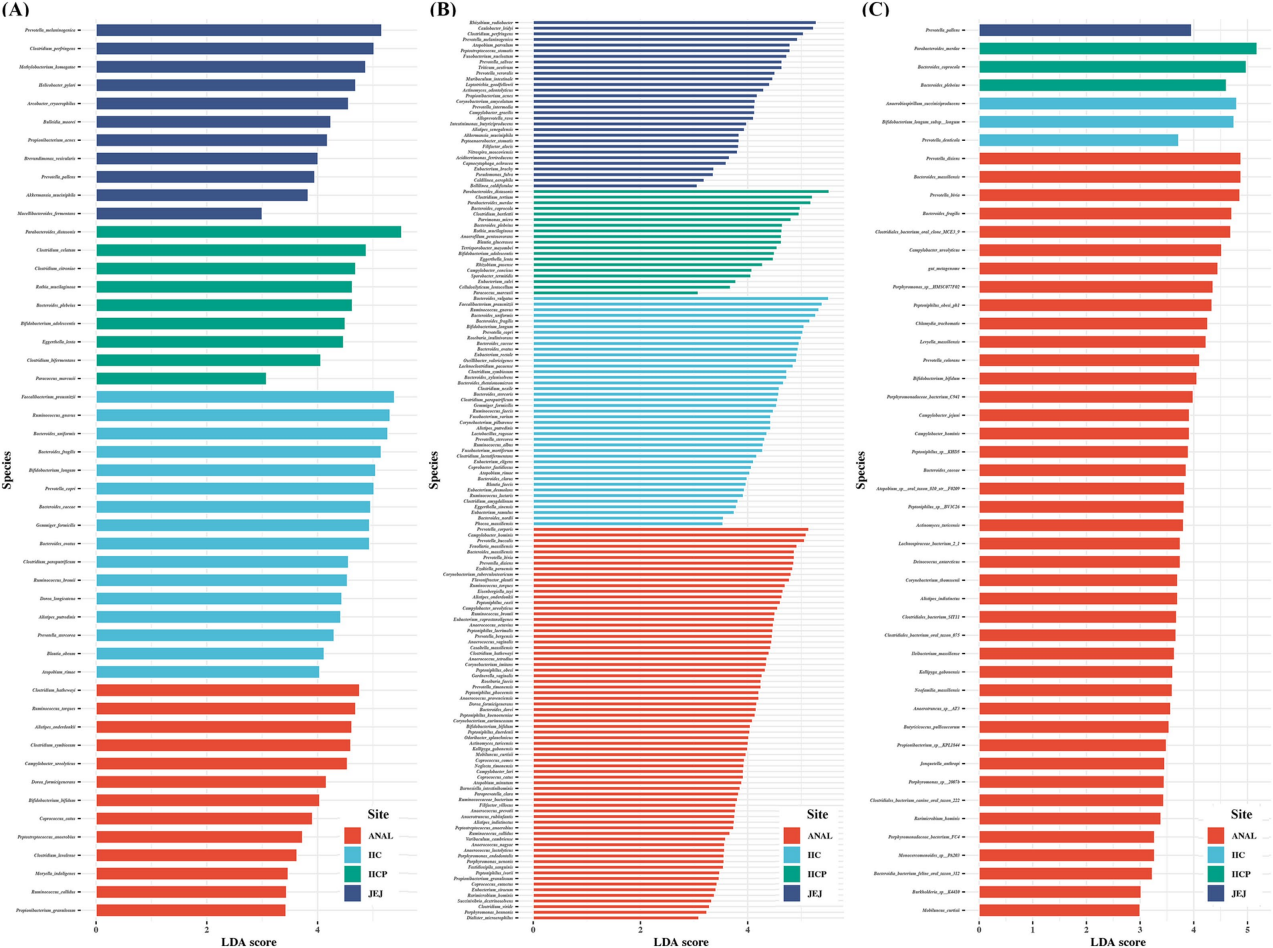
Comparisons of microbiota among various gut sampling sites that presented significantly different depending on reference database. **(A)** Greengene, **(B)** PacBio ASVs, **(C)** SILVA. The analysis was performed using linear discriminant analysis (LDA) and effect size analysis. LDA score > 3.0 are displayed.

Finally, LEfSe was applied to evaluate the differential analysis in bacterial species abundance among gut sampling sites using the taxa assigned by each reference DB. Despite analyzing the same raw data, the results demonstrated a clear difference in the identification of significant taxa depending on the reference DBs. PacBio identified significantly more species compared to the other two reference DBs. The number of significant taxa varied depending on the DB. Five species were found significant across all reference DBs: *Bacteroides caccae, B. fragilis, B. plebeius, Bifidobacterium bifidum*, and *Campylobacter ureolyticus*. Additionally, 30 species overlapped between the Greengenes and PacBio DBs, while 11 species overlapped between the SILVA and PacBio DBs. *Prevotella pallens* was identified as significant by both Greengenes and SILVA DBs. There were some unique taxa identified significant depending on the reference DB. The Greengenes DB found 11 unique species, PacBio identified 114 unique species, and SILVA detected 32 unique species. Interestingly, the Greengene DB identified 4 significant *Clostridium* species, while the SILVA DB identified 4 significant *Clostridiales* bacterium and PacBio DB identified 6 unique *Clostridium* species. Additionally, the PacBio DB identified several genera with multiple unique significant taxa, including 7 unique *Anaerococcus* species, 9 *Bacteroides* species, 5 *Corynebacterium* species, 8 *Eubacterium* species, 7 *Peptoniphilus* species, and 8 *Prevotella* species (Figure 6). Taken together, marked variations were observed in the identification of bacterial species depending on the reference DBs, with PacBio demonstrating highest number of unique and significant taxa, suggesting the importance of reference DB.

## Discussion

Sequencing of the 16S rRNA gene is a widely accepted standard for analyzing the taxonomic composition of bacterial communities (Woese and Fox, 1977). Extensive public databases (e.g., SILVA, Greengenes, RDP) facilitate taxonomic assignment. Optimizing reference databases is crucial for human microbiome studies to ensure accurate identification and classification of microbial taxa, thereby reducing the chances of misidentification or ambiguous results (Monika Balvočiūtė et al., 2017). Recent advancements in PacBio technology can generate highly accurate, long high-fidelity reads, offering single-nucleotide resolution (Wenger et al., 2019; Callahan et al., 2019). In this study, we tested whether 16S full-length sequencing data produced by PacBio could be used to construct a reference database and evaluated its application using Illumina V3-V4 targeted short read sequencing data in human microbiome studies.

To evaluate whether PacBio long-read sequencing data could be used to construct a microbiome reference database, we used oral microbiome data for testing. The oral microbiome typically has lower microbial diversity compared to the gut microbiome (Human Microbiome Project C, 2012) and has been extensively studied, resulting in well-characterized reference DBs (18). First, we plotted the number of ASVs obtained from an individual to determine the minimum number of samples required to represent a population. For an individual oral sample, the average ASV count was 247, ranging from 34 to 440. Since combining and resequencing samples was not feasible, we randomly combined samples to simulate mixtures. When 4, 8, 16, and 32 samples were randomly combined, the number of ASVs detected increased gradually with the number of samples in the group (Figure 1B). To assess their efficiency in classifying Illumina data, we constructed a BLAST reference database using the PacBio ASVs obtained from various combinations. A stand-alone BLAST search was performed against Illumina data, and the results were compared against eHOMD to obtain discrete statistics. BLAST operates by aligning query sequences to a database of sequences, identifying regions of similarity using a heuristic algorithm to find high-scoring sequence alignments quickly. It produces a list of sequences in the database that are most similar to the query sequence, along with alignment scores and statistics, including identical nucleotide length and percentage (Altschul et al., 1990; Camacho et al., 2009).

When comparing eHOMD and PacBio_4 (4 samples mixed), which had a similar number of reference counts, the proportion of high identity (>97%) was significantly higher using eHOMD, while the overall positively blasted (>90%) percentage of reads was over 95% for both. Generally, sequence identity of 97, 95, and 90% or less for 16S rRNA genes is considered distinctive for species, genera, and family, respectively (Yarza et al., 2014; Tindall et al., 2010). The combination that showed comparable high identity performance to eHOMD was PacBio_16 (16 samples mixed). Thus, a minimum of 4 samples was sufficient to determine 95% of reads at the family level, while at least 16 samples were required to determine 95% of reads at the species level. Given that PacBio_32 included nearly 6,000 ASVs compared to eHOMD’s 1,032 sequences, we tested whether the eHOMD could be enhanced by adding ASVs from PacBio_32. Specifically, we filtered Illumina reads that showed less than 97% identity against eHOMD but higher than 97% identity against PacBio ASVs. We identified 130 ASVs, and the database created by combining eHOMD with these 130 ASVs (eHOMD+Pac) demonstrated the highest taxonomic assignment performance (Figure 1C). Taken together, with sufficient samples, PacBio full-length sequencing data can be utilized to construct a reference DB from a scratch for oral microbiome study.

To investigate any discrepancies in microbiome coverage between the PacBio DB and eHOMD, a phylogenetic tree was constructed using PacBio_32 OTU sequences combined with eHOMD. Phylogenetic analysis, which can be used for biological classification (de Queiroz and Gauthier, 1994) and predicting characteristics of clonal populations and unstudied species (Pearson et al., 2009), revealed that most of the trees included sequences from both databases, suggesting that both databases cover similar taxa (Figure 2).

Given the substantial size difference between the PacBio_32 ASVs and eHOMD databases, we aimed to optimize the PacBio ASVs. One method to optimize the database is by constructing a phylogenetic tree, trimming closely related branches, and retaining the representative taxa (Mikula, 2018). To find the optimal condition, terminal branches were trimmed at various thresholds. When these trimmed ASVs were used to BLAST Illumina sequencing data, a negative correlation was observed between the threshold and identity outcome. With a trimming threshold of 0.0005, the number of ASVs in the BLAST reference database was reduced by 50%, while the BLAST search performance remained similar to that of the PacBio_32 ASVs (Figure 3F). Taken together, this approach allows for efficient database optimization while maintaining high taxonomic assignment accuracy.

To evaluate whether PacBio ASVs could be applied to other less-studied microbiomes, we tested them against gut microbiome data. The gut microbiome, particularly in the small intestine, presents unique challenges. The microbial community composition in the small intestine differs from that in fecal or oral samples, often containing a higher proportion of fastidious and less well-characterized bacteria, which complicates taxonomic identification (Thadepalli et al., 1979; Villmones et al., 2022). Obtaining samples from the small intestine typically requires invasive procedures such as endoscopy or intubation, which are more complex, costly, and uncomfortable for patients compared to non-invasive fecal or oral sample collection (Booijink et al., 2007). Additionally, the small intestine has a lower microbial biomass compared to the colon, making it more difficult to obtain sufficient microbial DNA for analysis (Hayashi et al., 2005). We constructed optimized gut microbiome reference DB using gut PacBio ASVs.

A pre-trained Naive Bayes classifier was prepared using the Greengene DB, SILVA DB, and gut PacBio ASVs. Gut Illumina V3-V4 paired-reads microbiome data from the ileum, jejunum, and anus were taxonomically assigned by each classifier. Unlike BLAST, the Naive Bayes classifier assigns taxonomy to rRNA sequences by calculating the probability of the sequence belonging to a particular taxon. It is fast and efficient for classifying large numbers of sequences and provides taxonomic assignments with confidence scores, which depend on the quality and comprehensiveness of the training DB (Wang et al., 2007). At the genus level, the overall relative abundance showed similar proportions regardless of the DB used (Figure 5A). At the species level, classifiers trained with Greengene and SILVA DBs assigned more than 50% of the operational taxonomic units (OTUs) as *Bacteroides*, while the classifier trained with the PacBio DB distinguished most OTUs to specific species. Moreover, some species were only identified by the PacBio DB-trained classifier. Our results support that a well-curated, microbiome-specific DB can improve the reliability of 16S sequencing analyses and taxonomic annotations (Ritari et al., 2015; Sierra et al., 2020). Taken together, an improvement in species assignment was observed when using the PacBio DB across all four gut microbiome samples compared to the other two DBs.

One of the primary purposes of microbiome studies is to discover biomarkers for diseases (Hajjo et al., 2022). Biomarker discovery can provide a deeper understanding of disease mechanisms (Cani, 2018) and can be applied to disease prediction and treatment (Veziant et al., 2021; Marcos-Zambrano et al., 2021). We applied LEfSe to evaluate the biomarker discovery efficiency using classifiers trained with various reference DBs. The choice of reference DB significantly impacted the identification of significant taxa. The PacBio reference DB identified significantly more species compared to the other reference DBs. Although further validation is necessary, having more candidate species increases the likelihood of identifying important taxa.

In addition, recent advancements in the accuracy of sequencing long DNA reads using Nanopore technology, particularly in homopolymer regions, may present a new potential method for preparing microbiome reference DBs (Mantas Sereika et al., 2022).

## Conclusion

In conclusion, full-length 16S rRNA sequencing data produced by PacBio can be used to construct an optimized microbiome reference database that demonstrates coverage and efficiency comparable to the well-established HOMD in oral microbiome studies. Applying these optimization methods to gut microbiome data indicated that this approach could be extended to other microbiomes, enhancing the accuracy of microbiome classification and improving biomarker discovery.

## Data Availability

The raw sequencing data have been retrieved from NCBI GenBank BioProject ID PRJNA1049979. For oral microbiome study, 32 samples were sequenced by Pacbio and 198 samples were sequenced by Illumina platform. For gut microbiome study, 45 samples were sequenced by Pacbio and 128 samples were sequenced by Illumina. Summary of sampling site and sample number is shown in Tables 1, 2.

## References

[ref1] Altschul S. F. Gish W. Miller W. Myers E. W. Lipman D. J. (1990). Basic local alignment search tool. J. Mol. Biol. 215, 403–410. doi: 10.1016/S0022-2836(05)80360-2 2231712

[ref2] Baker M. (2010). Next-generation sequencing: adjusting to data overload. Nat. Methods 7, 495–499. doi: 10.1038/nmeth0710-495

[ref3] Bolyen E. Rideout J. R. Dillon M. R. Bokulich N. A. Abnet C. C. Al-Ghalith G. A.. (2019). Reproducible, interactive, scalable and extensible microbiome data science using QIIME 2. Nat. Biotechnol. 37, 852–857. doi: 10.1038/s41587-019-0209-9, PMID: 31341288 PMC7015180

[ref4] Booijink C. C. Zoetendal E. G. Kleerebezem M. de Vos W. M. (2007). Microbial communities in the human small intestine: coupling diversity to metagenomics. Future Microbiol. 2, 285–295. doi: 10.2217/17460913.2.3.285, PMID: 17661703

[ref5] Boppana K. Almansouri N. E. Bakkannavar S. Faheem Y. Jaiswal A. Shergill K.. (2024). Alterations in gut microbiota as early biomarkers for predicting inflammatory bowel disease onset and progression: a systematic review. Cureus 16:e58080. doi: 10.7759/cureus.58080, PMID: 38741828 PMC11088963

[ref6] Buetas E. Jordan-Lopez M. Lopez-Roldan A. D'Auria G. Martinez-Priego L. De Marco G.. (2024). Full-length 16S rRNA gene sequencing by PacBio improves taxonomic resolution in human microbiome samples. BMC Genomics 25:310. doi: 10.1186/s12864-024-10213-5, PMID: 38528457 PMC10964587

[ref7] Callahan B. J. Wong J. Heiner C. Oh S. Theriot C. M. Gulati A. S.. (2019). High-throughput amplicon sequencing of the full-length 16S rRNA gene with single-nucleotide resolution. Nucleic Acids Res. 47:e103. doi: 10.1093/nar/gkz569, PMID: 31269198 PMC6765137

[ref8] Camacho C. Coulouris G. Avagyan V. Ma N. Papadopoulos J. Bealer K.. (2009). BLAST+: architecture and applications. BMC Bioinform. 10:421. doi: 10.1186/1471-2105-10-421, PMID: 20003500 PMC2803857

[ref9] Cani P. D. (2018). Human gut microbiome: hopes, threats and promises. Gut 67, 1716–1725. doi: 10.1136/gutjnl-2018-316723, PMID: 29934437 PMC6109275

[ref10] Cole J. R. Wang Q. Fish J. A. Chai B. McGarrell D. M. Sun Y.. (2014). Ribosomal database project: data and tools for high throughput rRNA analysis. Nucleic Acids Res. 42, D633–D642. doi: 10.1093/nar/gkt1244, PMID: 24288368 PMC3965039

[ref11] de Queiroz K. Gauthier J. (1994). Toward a phylogenetic system of biological nomenclature. Trends Ecol. Evol. 9, 27–31. doi: 10.1016/0169-5347(94)90231-3 21236760

[ref12] DeSantis T. Z. Hugenholtz P. Larsen N. Rojas M. Brodie E. L. Keller K.. (2006). Greengenes, a chimera-checked 16S rRNA gene database and workbench compatible with ARB. Appl. Environ. Microbiol. 72, 5069–5072. doi: 10.1128/AEM.03006-05, PMID: 16820507 PMC1489311

[ref13] Dewhirst F. E. Chen T. Izard J. Paster B. J. Tanner A. C. Yu W. H.. (2010). The human oral microbiome. J. Bacteriol. 192, 5002–5017. doi: 10.1128/JB.00542-10, PMID: 20656903 PMC2944498

[ref14] Dong T. Liang Y. Xie J. Fan W. Chen H. Han X. (2024). Integrative analyses identify opportunistic pathogens of patients with lower respiratory tract infections based on metagenomic next-generation sequencing. Heliyon 10:e30896. doi: 10.1016/j.heliyon.2024.e30896, PMID: 38765026 PMC11097057

[ref15] Hajjo R. Sabbah D. A. Al Bawab A. Q. (2022). Unlocking the potential of the human microbiome for identifying disease diagnostic biomarkers. Diagnostics 12:1742. doi: 10.3390/diagnostics12071742, PMID: 35885645 PMC9315466

[ref16] Hall M. Beiko R. G. (1849). 16S rRNA gene analysis with QIIME2. Methods Mol. Biol. 1849, 113–129. doi: 10.1007/978-1-4939-8728-3_8 30298251

[ref17] Haneishi Y. Furuya Y. Hasegawa M. Picarelli A. Rossi M. Miyamoto J. (2023). Inflammatory bowel diseases and gut microbiota. Int. J. Mol. Sci. 24:3817. doi: 10.3390/ijms24043817, PMID: 36835245 PMC9958622

[ref18] Hayashi H. Takahashi R. Nishi T. Sakamoto M. Benno Y. (2005). Molecular analysis of jejunal, ileal, caecal and recto-sigmoidal human colonic microbiota using 16S rRNA gene libraries and terminal restriction fragment length polymorphism. J. Med. Microbiol. 54, 1093–1101. doi: 10.1099/jmm.0.45935-0, PMID: 16192442

[ref19] He B. Cao Y. Zhuang Z. Deng Q. Qiu Y. Pan L.. (2024). The potential value of oral microbial signatures for prediction of oral squamous cell carcinoma based on machine learning algorithms. Head Neck 46, 1660–1670. doi: 10.1002/hed.27795, PMID: 38695435

[ref20] Human Microbiome Project C (2012). Structure, function and diversity of the healthy human microbiome. Nature 486, 207–214. doi: 10.1038/nature11234, PMID: 22699609 PMC3564958

[ref21] Ivica Letunic Bork P. (2021). Interactive tree of life (iTOL) v5: an online tool for phylogenetic tree display and annotation. Nucleic Acids Res. 49, W293–W296. doi: 10.1093/nar/gkab301 33885785 PMC8265157

[ref22] Jie Z. Xia H. Zhong S. L. Feng Q. Li S. Liang S.. (2017). The gut microbiome in atherosclerotic cardiovascular disease. Nat. Commun. 8:845. doi: 10.1038/s41467-017-00900-1 29018189 PMC5635030

[ref23] Katiraei S. Anvar Y. Hoving L. JFP B. van Harmelen V. Willems van Dijk K. (2022). Evaluation of full-length versus V4-region 16S rRNA sequencing for phylogenetic analysis of mouse intestinal microbiota after a dietary intervention. Curr. Microbiol. 79:276. doi: 10.1007/s00284-022-02956-9, PMID: 35907023 PMC9338901

[ref24] Li L. Wang Z. He P. Ma S. Du J. Jiang R. (2016). Construction and analysis of functional networks in the gut microbiome of type 2 diabetes patients. Genom. Proteom. Bioinform. 14, 314–324. doi: 10.1016/j.gpb.2016.02.005, PMID: 27746285 PMC5093780

[ref25] Mantas Sereika Kirkegaard R. H. Karst S. M. Michaelsen T. Y. Sørensen E. A. Wollenberg R. D.. (2022). Oxford Nanopore R10.4 long-read sequencing enables the generation of near-finished bacterial genomes from pure cultures and metagenomes without short-read or reference polishing. Nat. Methods 19, 823–826. doi: 10.1038/s41592-022-01539-7 35789207 PMC9262707

[ref26] Marcos-Zambrano L. J. Karaduzovic-Hadziabdic K. Loncar Turukalo T. Przymus P. Trajkovik V. Aasmets O.. (2021). Applications of machine learning in human microbiome studies: a review on feature selection, biomarker identification, disease prediction and treatment. Front Microbiol. 12:634511. doi: 10.3389/fmicb.2021.634511 33737920 PMC7962872

[ref27] Mikula O. (2018). Cutting tree branches to pick OTUs: A novel method of provisional species delimitation. bioRxiv. 419887. [Preprint].

[ref28] Monika Balvočiūtė Huson D. H. (2017). SILVA, RDP, Greengenes, NCBI and OTT — how do these taxonomies compare? BMC Genomics 18:114. doi: 10.1186/s12864-017-3501-4 28361695 PMC5374703

[ref29] Pearson T. Okinaka R. T. Foster J. T. Keim P. (2009). Phylogenetic understanding of clonal populations in an era of whole genome sequencing. Infect. Genet. Evol. 9, 1010–1019. doi: 10.1016/j.meegid.2009.05.014 19477301

[ref30] Quast C. Pruesse E. Yilmaz P. Gerken J. Schweer T. Yarza P.. (2013). The SILVA ribosomal RNA gene database project: improved data processing and web-based tools. Nucleic Acids Res. 41, D590–D596. doi: 10.1093/nar/gks1219, PMID: 23193283 PMC3531112

[ref31] Ritari J. Salojarvi J. Lahti L. de Vos W. M. (2015). Improved taxonomic assignment of human intestinal 16S rRNA sequences by a dedicated reference database. BMC Genomics 16:1056. doi: 10.1186/s12864-015-2265-y, PMID: 26651617 PMC4676846

[ref32] Satam H. Joshi K. Mangrolia U. Waghoo S. Zaidi G. Rawool S.. (2023). Next-generation sequencing technology: current trends and advancements. Biology 12:997. doi: 10.3390/biology12070997 37508427 PMC10376292

[ref33] Sczyrba A. Hofmann P. Belmann P. Koslicki D. Janssen S. Droge J.. (2017). Critical assessment of metagenome interpretation-a benchmark of metagenomics software. Nat. Methods 14, 1063–1071. doi: 10.1038/nmeth.4458, PMID: 28967888 PMC5903868

[ref34] Segata N. Izard J. Waldron L. Gevers D. Miropolsky L. Garrett W. S.. (2011). Metagenomic biomarker discovery and explanation. Genome Biol. 12:R60. doi: 10.1186/gb-2011-12-6-r60, PMID: 21702898 PMC3218848

[ref35] She J. J. Liu W. X. Ding X. M. Guo G. Han J. Shi F. Y.. (2024). Defining the biogeographical map and potential bacterial translocation of microbiome in human 'surface organs'. Nat. Commun. 15:427. doi: 10.1038/s41467-024-44720-6, PMID: 38199995 PMC10781665

[ref36] Sierra M. A. Li Q. Pushalkar S. Paul B. Sandoval T. A. Kamer A. R.. (2020). The influences of bioinformatics tools and reference databases in analyzing the human Oral microbial community. Genes (Basel) 11:878. doi: 10.3390/genes11080878, PMID: 32756341 PMC7465726

[ref37] Souza A. K. Zangirolamo A. F. Droher R. G. FGC B. Alfieri A. A. (2023). Carvalho da Costa M, et al. investigation of the vaginal microbiota of dairy cows through genetic sequencing of short (Illumina) and long (PacBio) reads and associations with gestational status. PLoS One 18:e0290026:e0290026. doi: 10.1371/journal.pone.0290026, PMID: 37611040 PMC10446230

[ref38] Stackebrandt E. Goebel B. M. (1994). Taxonomic note: a place for DNA-DNA Reassociation and 16S rRNA sequence analysis in the present species definition in bacteriology. Int. J. Syst. Evol. Microbiol. 44, 846–849. doi: 10.1099/00207713-44-4-846

[ref39] Thadepalli H. Lou M. A. Bach V. T. Matsui T. K. Mandal A. K. (1979). Microflora of the human small intestine. Am. J. Surg. 138, 845–850. doi: 10.1016/0002-9610(79)90309-X 389076

[ref40] Tindall B. J. Rossello-Mora R. Busse H. J. Ludwig W. Kampfer P. (2010). Notes on the characterization of prokaryote strains for taxonomic purposes. Int. J. Syst. Evol. Microbiol. 60, 249–266. doi: 10.1099/ijs.0.016949-0, PMID: 19700448

[ref41] Veziant J. Villeger R. Barnich N. Bonnet M. (2021). Gut microbiota as potential biomarker and/or therapeutic target to improve the Management of Cancer: focus on Colibactin-producing *Escherichia coli* in colorectal Cancer. Cancers (Basel) 13:2215. doi: 10.3390/cancers13092215, PMID: 34063108 PMC8124679

[ref42] Villmones H. C. Svanevik M. Ulvestad E. Stenstad T. Anthonisen I. L. Nygaard R. M.. (2022). Investigating the human jejunal microbiota. Sci. Rep. 12:1682. doi: 10.1038/s41598-022-05723-9 35102222 PMC8803847

[ref43] Wang Q. Garrity G. M. Tiedje J. M. Cole J. R. (2007). Naive Bayesian classifier for rapid assignment of rRNA sequences into the new bacterial taxonomy. Appl. Environ. Microbiol. 73, 5261–5267. doi: 10.1128/AEM.00062-07, PMID: 17586664 PMC1950982

[ref44] Wenger A. M. Peluso P. Rowell W. J. Chang P. C. Hall R. J. Concepcion G. T.. (2019). Accurate circular consensus long-read sequencing improves variant detection and assembly of a human genome. Nat. Biotechnol. 37, 1155–1162. doi: 10.1038/s41587-019-0217-9, PMID: 31406327 PMC6776680

[ref45] Woese C. R. Fox G. E. (1977). Phylogenetic structure of the prokaryotic domain: the primary kingdoms. Proc. Natl. Acad. Sci. USA 74, 5088–5090. doi: 10.1073/pnas.74.11.5088, PMID: 270744 PMC432104

[ref46] Yarza P. Yilmaz P. Pruesse E. Glockner F. O. Ludwig W. Schleifer K. H.. (2014). Uniting the classification of cultured and uncultured bacteria and archaea using 16S rRNA gene sequences. Nat. Rev. Microbiol. 12, 635–645. doi: 10.1038/nrmicro3330, PMID: 25118885

