# Supplementary material for: Optimizing microbiome reference databases with PacBio full-length 16S rRNA sequencing for enhanced taxonomic classification and biomarker discovery
Source: Front Microbiol. 2024 Nov 25;15:1485073. doi: 10.3389/fmicb.2024.1485073 (PMC11625778; doi:10.3389/fmicb.2024.1485073)
Supplement: Supplementary file 1 [file Supplementary_file_1.docx]

**Supplementary Figures**


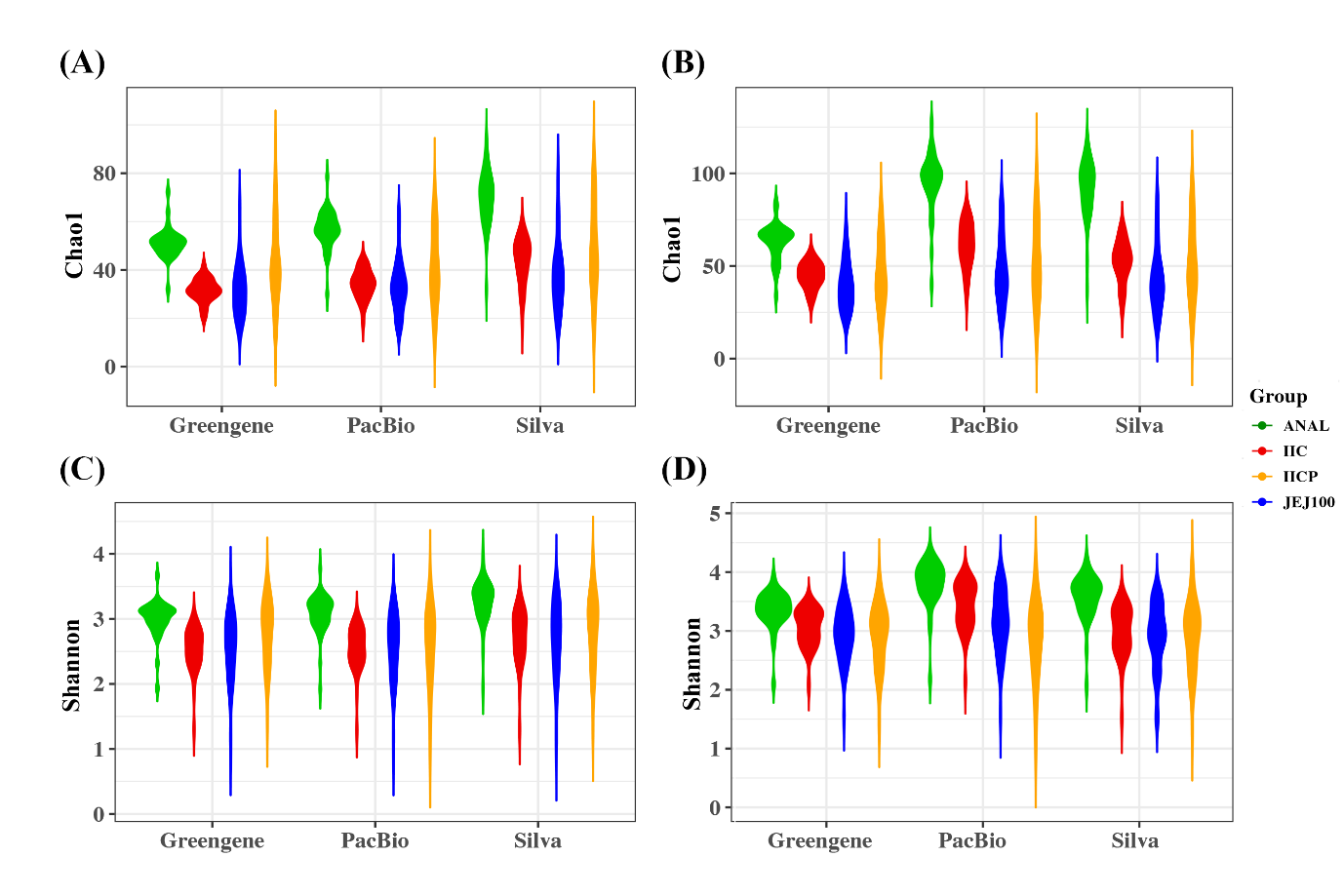


Fig. S1. Bacterial community comparisons among gut sampling sites at various taxonomic level. Alpha diversity was used to describe the microbial richness and evenness within samples using the Chao1 and Shannon index. (A) Chao1 index at Genus level, (B) Chao1 index at Species level, (C) Shannon index at Genus level, and (D) Shannon index at Species level.
